# Interleukin-1β and MicroRNA-146a as Prognostic and Diagnostic Markers of Systemic Lupus Erythematosus Complexity

**DOI:** 10.30699/ijp.2025.2049673.3394

**Published:** 2025-11-08

**Authors:** Saeed Mohammadi, Haider Fazel Hassan, Mojtaba Zare-Ebrahimabad, Fakhri Sadat Seyedhosseini, Ahmed Al-Harrasi, Yaghoub Yazdani

**Affiliations:** 1 Golestan Research Center of Gastroenterology and Hepatology, Golestan University of Medical Sciences, Gorgan, Iran; 2 Natural and Medical Sciences Research Center, University of Nizwa, Nizwa, Oman; 3 Department of Immunology, Faculty of Medicine, Golestan University of Medical Sciences, Gorgan, Iran; 4 Metabolic Disorders Research Center, Golestan University of Medical Sciences, Gorgan, Iran; 5 Department of Internal Medicine, School of Medicine, Golestan University of Medical Sciences, Gorgan, Iran; 6 Laboratory Sciences Research Center, Golestan University of Medical Sciences, Gorgan, Iran

**Keywords:** systemic lupus erythematosus, microRNA-146a, interleukin-1β, biomarker, diagnostic accuracy, lupus nephritis

## Abstract

**Background & Objective:**

Systemic lupus erythematosus (SLE) is a heterogeneous autoimmune disorder characterized by dysregulated autoantibody production and diverse clinical manifestations. Despite advances in research, the diagnosis and management of SLE remain challenging. This study evaluated plasma levels of interleukin-1β (IL-1β) and microRNA-146a (miR-146a) in patients with SLE and explored their potential as diagnostic and prognostic biomarkers.

**Methods:**

Blood samples were collected from 100 patients with SLE and 100 healthy controls. Patients with SLE were further classified into newly diagnosed (ND; n=50) and under treatment (UT; n=50) subgroups. Plasma IL-1β levels were quantified using ELISA, and circulating miR-146a expression was assessed by quantitative reverse transcription PCR.

**Results:**

Patients with SLE exhibited significantly higher plasma levels of IL-1β and miR-146a compared with healthy controls. ND patients demonstrated the highest concentrations of both biomarkers. Among patients with SLE, those with lupus nephritis (LN) showed markedly elevated IL-1β levels compared with those without LN. Longitudinal analysis during a 24-week follow-up indicated that higher baseline IL-1β levels were associated with an increased risk of LN development, supporting its potential prognostic relevance.

**Conclusion:**

IL-1β and miR-146a are elevated in patients with SLE, with IL-1β levels correlating with new-onset disease and LN development. These findings suggest that IL-1β and miR-146a may serve as useful biomarkers for diagnosing, monitoring, and predicting disease progression in SLE, although further validation is warranted.

## Introduction

Systemic lupus erythematosus (SLE) is a multisystem autoimmune disorder characterized by autoantibody production and immune-mediated organ damage. Common clinical manifestations include fatigue, fever, joint pain, skin rashes, and organ dysfunction, which complicate disease management due to the potential for severe complications (1). The economic burden of SLE is substantial, largely owing to costly treatments and loss of productivity (2). Prevalence rates vary globally, ranging from 20 to 150 cases per 100,000 in North America and Europe, with higher incidence reported among women and individuals of African or Hispanic descent. In the Middle East, prevalence differs across countries (3).

More than 80 genes have been implicated in SLE, most of which regulate immune responses. Environmental factors such as infections and ultraviolet (UV) radiation also contribute to disease onset and exacerbation (4). Current treatment strategies primarily aim to control symptoms and suppress disease activity. Corticosteroids and nonsteroidal anti-inflammatory drugs (NSAIDs) are used for inflammation and pain relief, while more severe cases often require immunosuppressants or biologics (5). Despite therapeutic progress, no curative treatment exists.

The pathogenesis of SLE involves both genetic susceptibility and epigenetic modifications (6). Mechanisms such as histone modification, DNA methylation, noncoding RNAs, and chromatin remodeling regulate immune-related genes without altering the DNA sequence (7). These epigenetic changes may provide opportunities for developing novel diagnostic tools and therapeutic strategies (8–10). Diagnosis and monitoring of SLE rely on established molecular biomarkers. According to the American College of Rheumatology (ACR), diagnostic criteria include clinical features and immunologic markers such as complement titers (C3, C4), anti–double-stranded DNA (anti-dsDNA) antibodies, antinuclear antibodies (ANA), inflammatory markers (CRP, ESR), and proinflammatory cytokines including interferon-α (IFN-α) and B-cell activating factor (BAFF) (11,12). However, conventional biomarkers have limitations, including low specificity and sensitivity, variability over time, high cost, and invasiveness, highlighting the need for new, reliable biomarkers (13).

Cytokine dysregulation is central to SLE pathogenesis, driving autoimmunity, chronic inflammation, tissue damage, and organ dysfunction (14). Elevated IL-6, IFN-α, TNF-α, and IL-17 levels are associated with heightened inflammatory responses, while decreased IL-10 may contribute to loss of immune regulation (15–17). Interleukin-1β (IL-1β), a proinflammatory cytokine, is increased in patients with SLE (18). IL-1β promotes B-cell activation, T-cell stimulation, and NLRP3 inflammasome activation, thereby contributing to disease progression (19). In animal models, blocking IL-1β signaling reduced disease activity and improved survival (20), and clinical trials with IL-1 receptor antagonists have shown promising results in patients with SLE (21). These findings suggest IL-1β may serve as a useful biomarker for assessing disease activity and severity (11), though further research is required.

MicroRNAs (miRNAs), small noncoding RNAs that regulate gene expression post-transcriptionally, play critical roles in immune regulation and autoimmunity (22). Several miRNAs, including miR-125a, miR-155, miR-223, miR-511-3p, miR-27a, and miR-146a, modulate immune responses (23,24). In SLE, reduced expression of miR-146a has been linked to enhanced proinflammatory signaling (25). miR-146a functions as an anti-inflammatory regulator by suppressing IRAK1/TRAF6-mediated NF-κB activation (26,27).

In this study, we aimed to quantify plasma levels of IL-1β and miR-146a in patients with SLE and healthy controls, examine their correlation, and assess their diagnostic and prognostic utility.

## Materials and Methods

### Participants and sampling strategy

This research study included 100 individuals diagnosed with SLE (PAT), equally comprising 50 newly diagnosed (ND) and 50 under-treatment (UT) patients. These participants were assigned from the Rheumatology Clinic at Sayyad Shirazi Hospital in Gorgan. Furthermore, 100 healthy individuals contributed as controls. A comprehensive set of criteria was used to ensure the eligibility of participants in both the SLE and control groups. Key inclusion criteria included a confirmed SLE diagnosis accomplished by a rheumatic disease specialist based on the ACR principles for SLE cases, and healthy status with no history of autoimmune disease for controls. The matching process of age, sex, and ethnicity covariates was performed for both groups. All enrollees presented authorized consent. The Committee of Ethics at Golestan University of Medical Sciences (GOUMS) in Gorgan, Iran, accredited the ethical practices, and we implemented the values outlined in the Declaration of Helsinki (28).

To minimize confounding factors, we excluded participants with other autoimmune diseases, active infections, pregnancy, chronic health conditions (such as chronic kidney disease, diabetes, cardiovascular disease), active malignancy, infectious diseases (including Hepatitis B/C, HIV), and individuals using medications or substances known to influence the markers under investigation (such as hormonal contraceptives, NSAIDs, immunomodulatory/ immunosuppressive drugs), as well as those who use tobacco or alcohol. We considered only those subjected to treatment for at least 6 months in the UT subgroup. Whole blood samples (5 mL) were collected from all participants and transported to the Research Central Laboratory at GOUMS for processing and analysis. Plasma was isolated from the whole blood, and stored at -80°C. The clinical data of all participants can be found in our previous publication (24).

### IL-1β cytokine measurement

The IL-1β plasma quantities were determined utilizing a commercially available enzyme-linked immunosorbent assay (ELISA) kit (ZellBio, Germany, cat. NO. RK00001-96). The evaluations were carried out as implied in the company's protocols. Optical density (OD) readings from the samples and standards were quantified utilizing a StatFax 3300 microplate reader (Awareness Technology, United States). Eventually, we used non-linear regression analysis to create standard curves and calculate the amounts of IL-1β in each sample, presented in picograms per milliliter (pg/mL).

### RNA purification, cDNA synthesis, and miR-146a expression evaluation

We extracted total RNA from plasma samples exploiting TRIzol reagent (Invitrogen, Germany), with minor modifications to the company's guidelines (29). We also measured RNA quality and concentration using a NanoDrop 2000 spectrophotometer (Thermo Scientific, USA). Then, we prepared cDNA from the extracted RNA (500 ng) using a reverse transcription kit (Cinnagen, Iran), after removing genomic DNA. The generated cDNA was saved at -20°C. Quantification of miR-146a expression was performed via SYBR Green-based qRT-PCR (Yekta Tajhiz Azma, Iran) using primers specifically designed for mature miR-146a. The forward primer sequence for miR-146a was 5'-GTCGTATCCAGTGCAGGGTCCGAGGTATTCGCACTGGATACGACCACTCCCCAT-3' and the reverse primer sequence was 5'-CCGGGTGTCGTGGAGTCG-3'. The adopted internal control was U6 small nuclear RNA, with the forward primer sequence 5'-GCTTCGGCAGCACATATACTAAAAT-3' and the reverse primer sequence 5'-CGCTTCACGAATTTGCGTGTCAT-3'. The PCR amplification was done in a Step One Plus unit (Applied Biosystems, USA). The relative expression of miR-146a was adjusted to U6 employing the 2^-ΔCt^ method.

### Statistical Assessments

Statistical analyses were performed using SPSS version 22 (IBM, USA) and GraphPad Prism version 8 (GraphPad, USA). For normally distributed data, comparisons between groups were conducted using independent-samples *t* tests, whereas nonparametric data were analyzed using Mann-Whitney *U* tests. Comparisons across three or more groups were performed using one-way analysis of variance (ANOVA) for normally distributed data and the Kruskal-Wallis test for non-Gaussian data.

Receiver operating characteristic (ROC) curve analysis was used to assess the diagnostic utility of IL-1β and miR-146a. Optimal cutoff values were determined using Youden’s index to maximize sensitivity and specificity (30). Logistic regression was applied to evaluate the predictive value of these biomarkers after 24 weeks of follow-up.

All statistical tests were two-tailed, with a significance threshold of *P* < .05 and a statistical power of 80%. Each analysis was performed in triplicate.

**Fig. 1:**
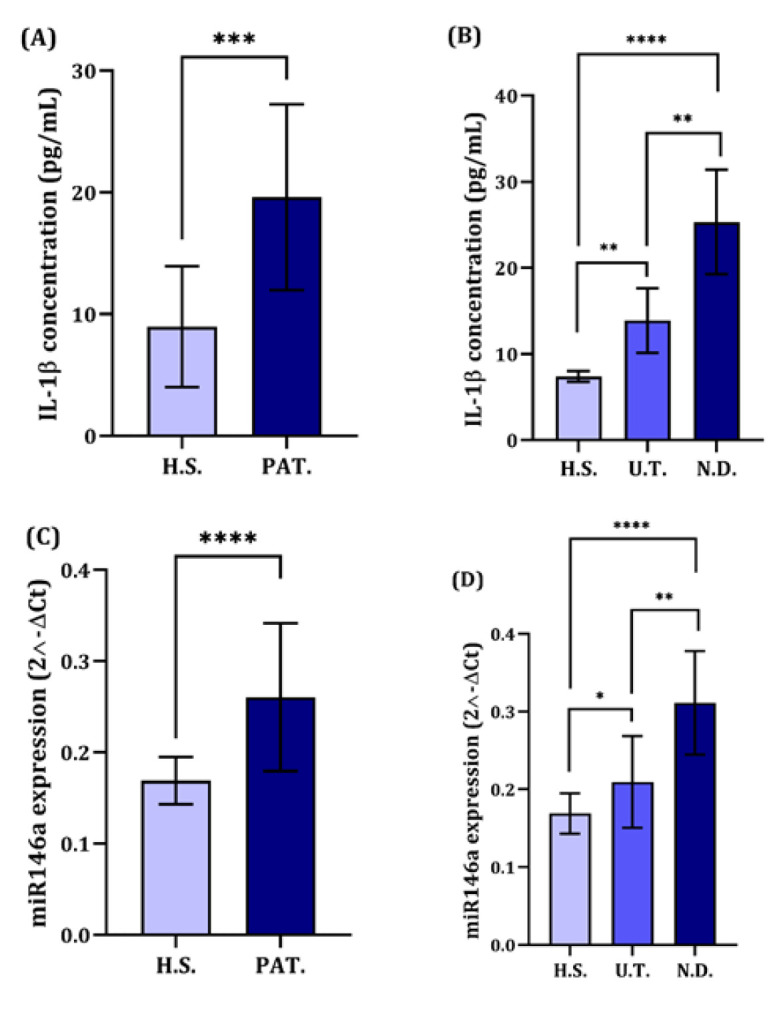
Elevated IL-1β and miR-146a Levels in SLE Patients.

## Results

### Augmented IL-1β and miR-146a Concentrations in SLE Patients

In this research, we inspected the plasma concentrations and levels of IL-1β and miR-146a among SLE cases versus non-diseased controls. Our experiments displayed that SLE patients had markedly elevated levels of IL-1β than controls (P < 0.001) (Figure 1A). Among the SLE subgroups, ND patients exhibited the highest IL-1β expression levels than the UT cases (P < 0.01) and healthy volunteers (P < 0.001) (Figure 1B). An independent samples t-test disclosed that SLE patients had significantly higher miR-146a expression levels than healthy individuals (P < 0.001) (Figure 1C). Moreover, newly diagnosed patients exhibited the most elevated levels of miR-146a compared to the UT subgroup (P < 0.01) (Figure 1D).

### The Effectiveness of IL-1β in Discriminating SLE Patients

**Fig. 2:**
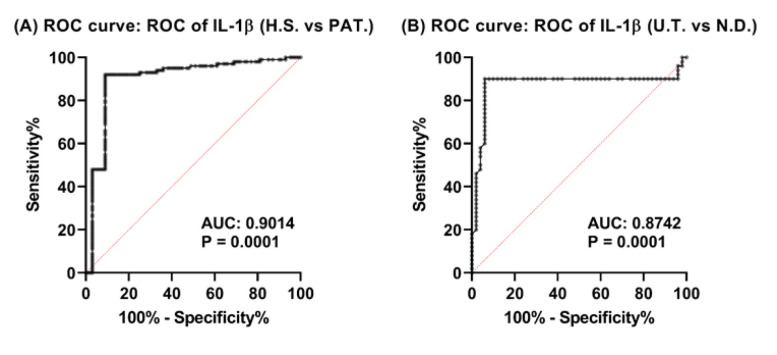
**Diagnostic Performance of IL-1β in Differentiating SLE Patients**. ROC curve for distinguishing PAT from HS, the AUC was 0.9014 (95% CI 0.8524 to 0.9503; P < 0.0001), with a sensitivity of 92.00% (95% CI 85.00% to 95.89%), specificity of 91.00% (95% CI 83.77% to 95.19%), and LR of 10.22. The calculated cut-off value was 12.49 (A). Differentiating ND patients from UT, the AUC was 0.8742 (95% CI 0.7888 to 0.9596; P < 0.0001), with a sensitivity of 90.00% (95% CI 78.64% to 95.65%), specificity of 94.00% (95% CI 83.78% to 98.36%), and LR of 15.00. The determined cut-off value was 20.01 (B). HS: Healthy subjects; PAT: Patients; UT: Under-treatment; AUC: Area under the curve; LR: Likelihood ratio; ND: Newly diagnosed.

We applied ROC curve analysis to assess IL-1β's diagnostic capability in distinguishing SLE patients from healthy volunteers, and ND patients from the UT subgroup. The plasma concentrations of IL-1β (HS vs. PAT) demonstrated an area under the curve (AUC) of 0.9014 (95% CI 0.8524 to 0.9503; P < 0.0001). We established the cut-off point at 12.49 with a sensitivity of 92.00% (95% CI 85.00% to 95.89%), specificity of 91.00% (95% CI 83.77% to 95.19%), and likelihood ratio (LR) of 10.22 (Figure 2A). For IL-1β (ND vs. UT), we obtained an AUC of 0.8742 (95% CI: 0.7888 to 0.9596; P < 0.0001). We set the cut-off value at 20.01, which granted a sensitivity of 90.00% (95% CI: 78.64% to 95.65%), a specificity of 94.00% (95% CI: 83.78% to 98.36%), and an LR of 15.00 (Figure 2B).

### Performance of miR-146a in Diagnosing SLE

The diagnostic utility of miR-146a in distinguishing SLE patients from HS and ND patients from the UT patients was assessed using ROC curve analysis. We discovered that miR-146a expression (HS vs. PAT) secured an AUC of 0.8568 (95% CI 0.8018 to 0.9117; P < 0.0001). The sensitivity was 80.00% (95% CI 71.12% to 86.66%), specificity was 74.00% (95% CI 64.63% to 81.60%), and the LR was 3.077. The cut-off threshold was defined at 0.1882 FC (Figure 3A). Concerning miR-146a (ND vs. UT), the AUC was 0.8800 (95% CI 0.8131 to 0.9469; P < 0.0001), demonstrating a sensitivity of 88.00% (95% CI 76.20% to 94.38%) and a specificity of 83.00% (95% CI 71.78% to 90.94%). The cut-off value was 0.2414 FC (Figure 3B).

### Expression of IL-1β in SLE Patients with Lupus Nephritis (LN) Compared to Non-Nephritis Cases

**Figure 4:**
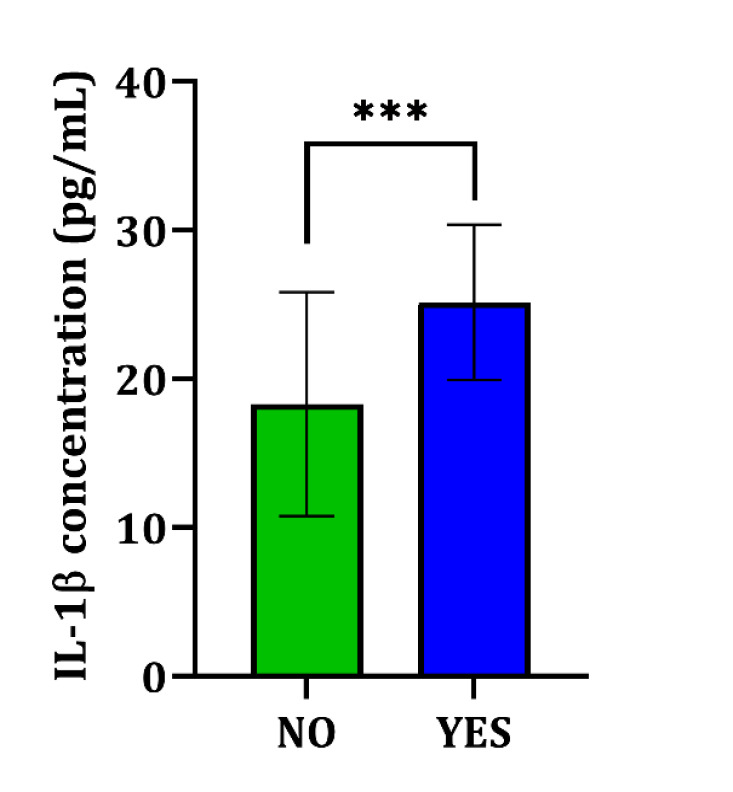
IL-1β expression was elevated in SLE patients with Lupus Nephritis (LN). To analyze the differences in IL-1β expression between patients with and without LN, we applied a t-test for comparative analysis. The results indicated a statistically significant difference in IL-1β expression levels, with patients with LN exhibiting notably higher levels (P = 0.001). This suggests that IL-1β could be a good marker for the presence of LN in SLE patients. Significance level is denoted as ***P < 0.001, derived from an independent samples t-test.

To analyze IL-1β expression differences in SLE patients with and without LN, we applied a t-test comparative method. There was a significant difference in IL-1β expression levels between SLE patients with LN and without, showing higher levels in patients with LN (P = 0.001) (Figure 4).

**Fig, 3:**
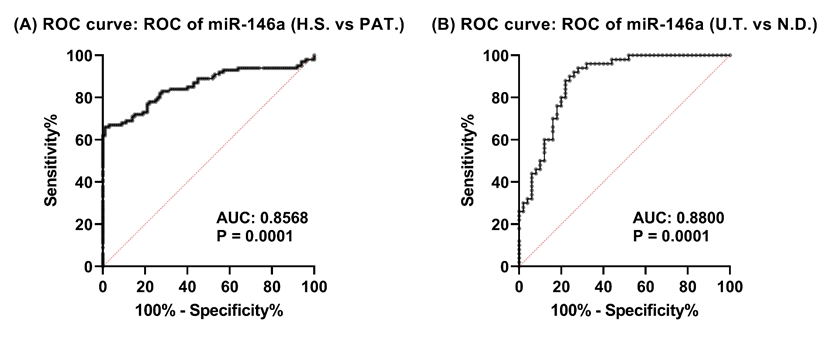
Diagnostic Performance of miR-146a. ROC curve illustrating the ability of miR-146a to distinguish PAT from HS, the AUC was 0.8568 (95% CI 0.8018 to 0.9117; P < 0.0001) (A). Differentiating ND patients from UT, the AUC was 0.8800 (95% CI 0.8131 to 0.9469; P < 0.0001). HS: Healthy subjects; PAT: Patients; AUC: Area under the curve; UT: Under-treatment; ND: Newly diagnosed.

### Prognostic Potential of IL-1β in Predicting LN

**Fig. 5:**
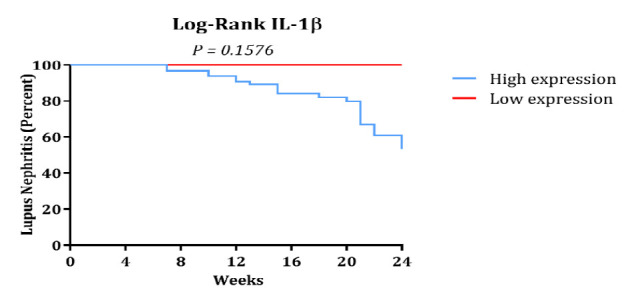
. Prognostic Potential of IL-1β in Predicting Lupus Nephritis. Kaplan-Meier curves illustrating the cumulative incidence of lupus nephritis based on IL-1β levels in SLE patients. Although patients with higher IL-1β levels showed a trend toward increased LN risk, this association was not statistically significant (P = 0.1576), and results should be interpreted with caution.

A follow-up period of 24 weeks was used to scrutinize the IL-1β’s prognostic value for predicting the development of LN in SLE patients. We analyzed the cumulative incidence of LN based on IL-1β levels using log-rank Mantel-Cox analysis and Kaplan-Meier curves. Within the cohort of 100 SLE patients, 19 individuals developed LN during the evaluation time frame. A median IL-1β level of 12.49 was identified as the optimal cut-off point for stratifying patients. Patients with IL-1β levels exceeding the threshold showed significantly greater risk of developing LN compared to those below the cut-off (Figure 4). Despite the follow-up, the statistical findings revealed no significant association between IL-1β levels and the progression to LN (P = 0.1576). The hazard ratio (HR) for LN in patients with IL-1β levels above the cut-off was 3.128 (95% CI 0.6433 to 15.21), suggesting a trend towards increased risk but lacking statistical confirmation (Figure 5).

## Discussion

Recent research has increasingly emphasized the identification of reliable biomarkers with both diagnostic and prognostic value in autoimmune diseases (11,31). In this study, we evaluated plasma levels of IL-1β and miR-146a in patients with SLE (both newly diagnosed and under treatment) compared with healthy controls. Our primary aim was to assess their diagnostic potential in distinguishing patients with SLE from healthy subjects and in differentiating patient subgroups (newly diagnosed vs under treatment). We also examined whether IL-1β could predict the development of lupus nephritis (LN), a major complication of SLE (26).

We observed that IL-1β levels were significantly elevated in patients with SLE compared with healthy controls. Among subgroups, newly diagnosed patients had the highest IL-1β expression, consistent with findings by Yongkang et al, who reported strong IL-1β expression in RF+IgE+ SLE patients, suggesting its role in disease pathogenesis (19). Similarly, Umare et al found elevated IL-1β levels in patients with SLE, correlating with disease activity (SLEDAI scores) and implicating IL-1β as an inflammatory mediator during active disease (32). Zhan et al demonstrated that IL-1β levels were significantly lower in primary immune thrombocytopenia than in SLE-associated thrombocytopenia and other SLE groups, suggesting its potential role in differentiating hematologic complications (33). Lovato et al showed that skin biopsies from cutaneous lupus erythematosus (CLE) patients who later developed SLE had higher IL-1β expression, highlighting its possible predictive role in CLE-to-SLE progression (34).

Contradictory results have also been reported. Mende et al found no significant difference in IL-1β serum concentrations between patients with SLE and healthy controls, nor correlation with disease activity. This discrepancy may reflect study design limitations, including lack of matching by age, sex, or ethnicity (19). In animal models, Loftus et al reported worsened renal pathology in female mice lacking IL-1β, despite no effect on overall survival or autoantibody production, suggesting complex roles of IL-1β in disease modulation (35). To our knowledge, no prior study has systematically examined the diagnostic and prognostic roles of IL-1β and miR-146a across SLE subgroups, making our analysis the first to address this gap.

We also found that plasma levels of miR-146a were significantly elevated in patients with SLE, with the highest levels observed in newly diagnosed patients. To date, no study has assessed plasma miR-146a expression in SLE subgroups. El-Akhras et al investigated polymorphisms of miR-146a and found that although single nucleotide variants were not directly associated with SLE susceptibility, the AG genotype and G allele of rs57095329 were linked to increased disease risk (36). Zhu et al reported decreased miR-146a expression in LN patients, which correlated with LN activity, increased IL-1β levels, and greater risk of ESRD progression and LN recurrence (37). Although our study found elevated plasma miR-146a in patients with SLE and in the newly diagnosed subgroup, we did not observe significant differences in patients with LN, which is partly consistent with Zhu et al’s findings. Similarly, Pauley et al observed upregulated miR-146a in PBMCs of rheumatoid arthritis patients, underscoring its role in autoimmune pathophysiology (38).

Taken together, our findings support IL-1β as a strong diagnostic marker for distinguishing patients with SLE from healthy individuals, as well as for differentiating newly diagnosed from treated patients. miR-146a also showed acceptable diagnostic performance in these contexts. However, given the relatively small sample size, there is a risk of overfitting in ROC threshold estimation, and validation in larger, multicenter cohorts is required.

We further observed that IL-1β levels were higher in patients with LN than in those without LN, suggesting its potential role in LN development. However, IL-1β concentrations above a cutoff of 12.49 did not achieve statistical significance in predicting LN progression during the 24-week follow-up, although an elevated risk trend was evident. The lack of statistical significance may be due to limited power, as only 19 LN events were recorded. The wide 95% CI for the hazard ratio (0.64–15.21) reflects this uncertainty. Additionally, we did not evaluate miR-146a differences between LN and non-LN patients, representing an area for future research.

Our study has several limitations. The cross-sectional design limits causal inference, and the 24-week follow-up may be insufficient to capture long-term LN progression. Despite applying exclusion criteria, residual confounding from medication heterogeneity and comorbidities may persist. Generalizability is limited due to single-center sampling. Future multicenter longitudinal studies with larger, diverse populations are needed to validate and extend our findings.

## Conclusion

In summary, plasma levels of IL-1β and miR-146a were elevated in patients with SLE, with IL-1β showing strong diagnostic value for identifying early-stage disease and miR-146a demonstrating potential for distinguishing subgroups. IL-1β levels were also associated with LN, suggesting possible prognostic utility. However, larger longitudinal cohort studies are required to confirm the clinical applicability of these biomarkers in diagnosing, monitoring, and predicting disease progression in SLE.

## Data Availability

The datasets used and/or analyzed during the current study are available from the corresponding author on reasonable request.
